# The digital transformation of medicine can revitalize the patient-clinician relationship

**DOI:** 10.1038/s41746-018-0060-2

**Published:** 2018-09-20

**Authors:** Haider J. Warraich, Robert M. Califf, Harlan M. Krumholz

**Affiliations:** 1 Department of Medicine, Cardiology Division, Duke University School of Medicine, Durham, NC USA; 2 Duke Clinical Research Institute, Durham, NC USA; 3 Duke Forge, Durham, NC USA; 4 Department of Medicine, Stanford University, Palo Alto, CA USA; 5 Verily Life Sciences, South San Francisco, CA USA; 6 Section of Cardiovascular Medicine, Department of Medicine, Yale University School of Medicine, New Haven, CT USA

**Keywords:** Health care, Ethics, Health care, Ethics

## Abstract

Health professionals within the medical community feel that the principles of humanism in medicine have not been a point of emphasis for information and computer technology in healthcare. There is concern that the electronic health record is eroding the patient-clinician relationship and distancing clinicians from their patients. New analytic technologies, on the contrary, by taking over repetitive and mundane tasks, can provide an avenue to make medical care more patient-centered by freeing clinicians’ time, and the time of the whole clinical care team, to engage with patients. Technology such as advanced speech recognition that optimizes clinicians’ workflow could revitalize the patient-clinician relationship and perhaps also improve clinician well-being. Digital phenotyping can gain invaluable additional data from patients using technology that is already used for personal reasons by the majority of patients. The digital transformation of healthcare has the potential to make healthcare more humane and personalized, however, several important steps are needed to avoid the pitfalls that have come with prior iterations of information technology in medicine such as a heightened emphasis on data security and transparency. Both patients and clinicians should be involved from the early stages of development of medical technologies to ensure that they are person-centric. Technologists and engineers developing healthcare technologies should have experiences with the delivery of healthcare and the lives of patients and clinicians. These steps are necessary to develop a common commitment to the design concept that technology and humane care are not mutually exclusive, and in fact, can be symbiotic.

Humanism in medicine is characterized by a respectful and compassionate relationship between physicians and other members of the clinical team and patients.^1^ Health professionals within the medical community, however, feel that the principles of humanism in medicine have not been a point of emphasis for information and computer technology in healthcare, a view shaped in large part due to the way by the electronic health record (EHR) has been developed and implemented.^2^ While the introduction of the EHR has advanced the use of standards of clinical care, provided the ability to make records available across clinicians and granted the ability to access aggregate data to gain insights into better patterns of care, there is concern that by taking up a disproportionate amount of clinicians’ time, it is eroding the patient-clinician relationship.^2–4^ In a survey of 15,285 physicians across 25 specialties, most respondents felt that the EHR slowed down their workflow, reduced the amount of time they spent with their patients and the amount of patients they could see.^4^

New analytic technologies, on the contrary, by taking over repetitive and mundane tasks, can provide an avenue to make medical care more patient-centered by freeing the healthcare teams’ time to engage with patients. Furthermore, predictive analytics, which enable personalized estimates of risk and benefit, are intrinsically more attuned to individual differences than many current guidelines that are based on the average experiences of large groups of patients. However, for technology to enrich rather than undercut the patient-clinician relationship, medical humanism will need to be at the core of the design thinking behind emerging technologies and software. And more importantly, considerable effort will be needed to plan for how the technology improves the quality of human interactions rather than merely focusing on efficiency, both among team members and with patients and their families.

If humanistic design thinking is emphasized, technology has the potential to recapture the promise of both more effective and efficient healthcare and allow clinicians to be more attentive to their patients. If employed effectively, advances in predictive analytics and information technology could be used to improve clinical workflow, enhance our understanding of patient’s emotional state and human needs, and lead to a new era of humanism in medicine.

The patient-clinician interaction, whether in-person or through other means, has become increasingly complex.^5^ However, while the clinic visit in general has increased in duration contrary to general perception, the lengthening of the visit has been outpaced by the increase in the complexity of the health and medical problems patients and their families must consider.^5^ Clinicians typically spend more time practicing “desktop medicine” than spending face-to-face time with patients in the outpatient setting.^6^ On average, two-thirds of time spent practicing desktop medicine is used to write progress notes, while the rest of the time is used for telephone encounters, sending secure messages and writing prescriptions.^6^ In the inpatient setting too, an increasing proportion of time is spent on documentation over time.^7^ The reduction in the time of interactions between patients and their clinicians means that the period required to build a trusting relationship is also decreasing, a factor that is known to lead to increasing burnout among physicians.^8^

Technologies that optimize clinicians’ workflow and helps them spend more time with patients, whether face-to-face or through other means, could revitalize the patient-clinician relationship and perhaps also improve clinician well-being. Research suggests that errors are common in the EHR, and that seven out of every hundred words transcribed using conventional dictation technology are incorrect, requiring manual review and correction.^9^ Advanced speech recognition tools can be used to more accurately transcribe medical encounters between clinicians, patients and caregivers in real time during the clinic visit.^10^ Such an advance would save the healthcare team’s time, provide information untainted by recall bias, increase transparency and allow the patient and their caregivers’ conversations to become a part of the EHR. This technology could also be combined with speech emotion recognition systems, which can provide even greater insight into what patients and clinicians might be feeling that is not captured by the content of their dialogues.^11^ Data collected from these conversations could not only be used for record keeping and billing but could drive the next generation of research into shared decision making, informed consent, explicit and implicit biases, and could provide feedback to clinicians on improving their connection with patients. However, emphasis needs to be placed on developing and testing technologies that simplify communications among different members of the clinical team and minimize redundant documentation that can clutter the EHR.

The current medical framework largely restricts data collection to face-to-face meetings, limiting not only time for relationship building, but also the opportunity to leverage important information that patients can share with their clinicians. Digital phenotyping, the “measuring of behavior from smartphone sensors, keyboard interaction, and various features of voice and speech,” is a means to gain invaluable additional data from patients using technology that is already used for personal reasons by the majority of patients.^12^ Digital phenotyping has already been used to diagnose a wide range of mood disturbances, yielding invaluable information that would otherwise be difficult to obtain using conventional means. Such additional insights into patients’ well-being that are automatically generated and processed can foster a richer understanding of patients’ lives and provide evidence that is more directly relevant to them, a key to developing a strong patient-clinician relationship. Furthermore, the past decade has seen dramatic improvements in automated facial expression recognition software, and these techniques have already been used in such diverse settings such as detecting deception, racial prejudice, and operator fatigue.^13^ Automated facial expression analytics has also been used to predict future behavior, including the likelihood of a driver being involved in a car accident, online shoppers’ likelihood of buying specific products, and predicting whether individuals are gaining mastery of specific tasks.^13^

The key to the development of effective therapeutic relationships between patients and clinicians is understanding both verbal and non-verbal cues of emotions that patients express. Digital phenotyping has the potential to vastly improve this understanding and, therefore, the use of machine learning to decipher large-scale differences in human expression at scale can provide insights that can be tailored to the individual. Information garnered through these techniques could be used to predict and potentially mitigate future behaviors such as medication adherence, substance abuse, and effective behavioral and lifestyle changes. These outputs could also be imported into a data environment that includes the traditional EHR and sources from everyday life to provide even richer predictive information about patients. Such additional information could help better align patients and the medical team in their common goal of improving health and well-being.

For the digital transformation of medicine to build stronger bridges between healthcare teams and patients, there needs to be a heightened emphasis on data security and transparency. If patients cannot trust that their data will be secure, transparent and accessible to them, patient data collection might increase suspicion of healthcare systems rather than enhance trust. Simply providing technological advancement with no societal oversight to ensure that powerful tools are used to improve well-being carries considerable risk as evidenced by the recent use of personal data to create dissension and targeted misinformation.^14^ On the other hand if patients, families and clinicians operated with transparent rules and careful oversight of use of data, the current cumbersome EHR could be transformed into a larger data repository that served as a useful joint source of information to be used to empower human relationships, and the prevention and treatment of disease. These technologies can also increase opportunities for clinicians to learn from their patients about their health, and could engage them in additional opportunities to explore new frontiers in how patients and clinicians interact. However, the need to maintain privacy and transparency with full permission from patients is paramount as we move forward. Informed consent should make it explicitly clear what purposes data collected from patients will serve. It should also be clear how products will be designed, or inputs gathered from patient data, for the purpose of commercialization, if that is the intent.

If technologies are designed specifically to be linguistically and culturally adaptive, in some instances virtual interfaces can even exceed the humanistic capabilities of human beings. This has been demonstrated in behavioral counseling comparing ‘relational agents’ with human counselors regarding promoting positive health behaviors and virtual nurses in patients with low health literacy.^15,16^ Such virtual interfaces such as visual chat bots and other relational agents can add to face-to face interactions between the healthcare team and patients. However, a lack of humanistic design can have deleterious consequences as demonstrated by mathematical algorithms developed to predict criminality, which demonstrate bias against African Americans.^17^ Therefore, engineers and data scientists who develop such technologies should receive specific training regarding the ethical implications and unintended consequences of medical technologies.

The digital transformation of healthcare has the potential to make healthcare more humane and personalized, however, several important steps are needed to avoid the pitfalls that have come with prior iterations of information technology in medicine. Both patients and clinicians should be involved from the early stages of development of medical technologies to ensure that they are person-centric. Emphasis needs to be placed on simplifying communication among members of the healthcare team. Technologists and engineers developing healthcare technologies should have experiences with the delivery of healthcare and the lives of patients and clinicians. These steps are necessary to develop a common commitment to the design concept that technology and humane care are not mutually exclusive, and in fact, can be symbiotic. Technology, if harnessed and developed correctly, can emphasize presence, rather than distancing providers from their patients.
